# *In silico* molecular and morphological analysis of rice blast resistant gene *Pi-ta* in Sri Lankan rice germplasm

**DOI:** 10.1186/s43141-021-00239-7

**Published:** 2021-10-21

**Authors:** Suvanthini Terensan, H. Nishadi S. Fernando, J. Nilanthi Silva, S. A. Chandrika N. Perera, Nisha S. Kottearachchi, O. V. D. S. Jagathpriya Weerasena

**Affiliations:** 1 Institute of Biochemistry Molecular Biology and Biotechnology, University of Colombo, Colombo, Sri Lanka; 2 Regional Rice Research and Development Centre, Bombuwala, Sri Lanka; 3 Department of Agricultural Biology, Faculty of Agriculture, University of Peradeniya, Peradeniya, 20400 Sri Lanka; 4 Department of Biotechnology, Faculty of Agriculture and Plantation Management, Wayamba University of Sri Lanka, Makandura, Sri Lanka

**Keywords:** Amino acid polymorphism, LRD region, *Magnaporthe oryzae*, Molecular breeding, R genes

## Abstract

**Background:**

*Pi-ta* is a major blast resistant gene, introgressed from *indica* rice varieties. In this study, diversity of the *Pi-ta* gene of 47 Sri Lankan rice accessions was studied by bioinformatics, and the results were validated with molecular and disease reaction assays. Sequences of rice accessions at the locus Os12g0281300 were retrieved from Rice SNP-Seek Database, and the coding sequence of reference *Pi-ta* gene of cultivar *Tetep* (accession no. GQ918486.1) was obtained from GenBank. Comparisons were made at nucleotide, amino acid, and protein structure level, and the 3D models predicted using Phyre2 software were superimposed using TM-align software.

**Results:**

*In silico* analysis revealed that 10 accessions possessed resistant allele of the *Pi-ta* gene. The remaining accessions recorded high polymorphism in the leucine-rich domain resulting in 9 allele types, leading to single–amino acid substitutions at 27 different positions including a functional mutation of alanine to serine at the 918th amino acid position. None of the genotypes led to truncations in the amino acid sequence. The *in silico* analysis results were validated on 23 accessions comprising resistant and susceptible genotypes and another 25 cultivars from Northern Sri Lanka, by molecular assay using YL183/YL87 and YL155/YL87 resistant and susceptible allele-specific markers. Resistance of *Pi-ta* gene for the causal fungus, *Magnaporthe oryzae*, was further validated through pathogenicity assay.

**Conclusion:**

The *Pi-ta* gene, especially the LRD region, revealed significant variations within Sri Lankan rice cultivars leading to high levels of resistance against blast. This information would be highly useful in breeding programmes for resistance against rice blast.

**Supplementary Information:**

The online version contains supplementary material available at 10.1186/s43141-021-00239-7.

## Background

Rice is the staple diet for more than half of the world’s population. Rice blast, caused by the fungus *Magnaporthe oryzae,* is generally considered to be the most devastating rice disease, posing serious threats for cultivations across the world. Changing climatic conditions, and the highly variable nature of the pathogen have resulted in unpredictable and common blast epidemics failing disease management programmes. The most effective way to achieve durable disease resistance is the utilization of cultivars possessing disease-resistant genes (R genes) in breeding.

To date, 27 blast resistant genes have been cloned and characterized [1]. Among them, the *Pi-ta* gene is reported to be effective in combating the blast causing fungus [2–4]. This is a single copy gene, clustered at the centromere of chromosome 12 of rice. *Pi-ta* gene encodes 928 amino acids which contain the nucleotide-binding site (208–527), a conserved internal hydrophobic domain (407–418), leucine-rich domain (586–928), and four potential glycosylation sites (339, 556, 654, 838). The leucine-rich domain (LRD) in *Pi-ta* differs from the typical leucine-rich repeat (LRR) of other R genes due to the additional leucine repeats compared with LRR. Both LRD and LRR are found in the C-terminal region which is generally involved in the pathogen recognition [5]. *Pi-ta* / *Avr-Pita* is a well-studied ligand/receptor model where *Pi-ta* protein acts as a receptor, binds elicitor molecule *Avr*-*Pita* leading to defense response of the plant. The presence of a single amino acid polymorphism, serine instead of alanine, at the position of the 918th amino acid in the LRD region of the *Pi-ta* protein impairs the binding recognition with *Avr* gene of *M. oryzae* leading the pathogen to lose the ability to infect the plant [2]. This unique feature provides an opportunity to investigate the resistant *Pi-ta* gene in rice germplasm which is highly useful in view of the huge potential of utilizing the *Pi-ta* gene in managing the devastating blast disease of rice worldwide.

Generally, *indica* rice cultivars are a rich source of blast resistant genes with 51% of resistant genes which are already identified [6]*. Pi-ta gene* has also been introgressed from *indica* cultivars *(Tetep* and *Tadukan*) to other cultivated rice varieties ([7–9]). However, identification of new donors for blast resistance is a prerequisite to improve the germplasm of any country [10], because, specific varieties adopted for variations in cultivation patterns, seasons, etc., are preferred in producing varieties specific to each region [11]. This emphasizes the need to search for donors of specific origin for the management of blast. Sri Lankan rice cultivation is entirely with *indica* rice varieties, and thus there may be valuable resistant R gene sources among the cultivated varieties. The experimental screening of R genes is tedious and costly, but the utilization of bioinformatics tools provides a better option for developing countries to study the availability of resistant genes in a large number of samples with subsequent confirmation by molecular marker-based experiments for potential candidates [12]. Further, proper identification of R genes in diverse elite germplasm through DNA markers is a crucial step in confirming the precision in the exploitation of R gene in marker-assisted selection (MAS) in different rice breeding programmes [13].

Identifying the R genes in the local germplasm will be more useful in identifying suitable resistant cultivars which are also preferred by the consumers. However, the genetic data of the local cultivars are not available in any public domain. This report describes the first attempt of studying the variations of the *Pi-ta* gene using a combination of assays such as molecular marker analysis, 3D structural modelling, and pathogenicity assay for the Sri Lankan accessions. Accordingly, in the current study, we studied the diversity of *Pi-ta* gene mainly in the LRD region, in 47 Sri Lankan rice accessions through *in silico* analysis and the results were confirmed by disease reaction and molecular marker assay. This validated method was applied to detect the resistant *Pi-ta* gene in 25 preferred cultivars which are commonly cultivated in the Northern Province of Sri Lanka.

## Methods

### *In silico* analysis

#### Sequence retrieval

A complete *Pi-ta* gene sequence (7295 bp) of 47 Sri Lankan rice accessions (enlisted in the supplementary table 4) was retrieved from the Rice SNP-Seek Database of the International Rice Research Institute (https://snp-seek.irri.org/) by giving the following genotype query options; chromosome/ contig number 12, locus position of *Pi-ta* gene Os12g0281300 which was obtained from Oryzabase (https://shigen.nig.ac.jp/rice/oryzabase/gene/detail/947), and the reference sequence was Nipponbare (japonica).

A complete sequence of wild-type *Pi-ta* gene (*wPi-ta*), of *Oryza sativa* cultivar indica Tetep (GenBank accession number GQ918486.1), was downloaded from GenBank database (https://www.ncbi.nlm.nih.gov/genbank/). The coding sequence of the *Pi-ta* gene was considered as the region of interest for this analysis. The gene consists of two exons with the lengths of 944 bp and 1845 bp [14], and total length of the coding sequence was 2789 bp.

#### Comparison of nucleotide and amino acid sequences

Sequence variation of the accessions was studied by comparing the two exons of each accession with the wild-type *Pi-ta* (*wPi-ta*) of *Tetep* exons using Clustal W multiple alignment program in Bioedit Sequence Alignment Editor Version 7.2.5. Nucleotide polymorphisms among aligned sequences were noted.

The ORF finder tool of NCBI was used to derive the open reading frames of the exon sequences with single-nucleotide polymorphisms in the alignment to find out any possible truncations in coding frames. The corresponding amino acid sequences derived from the ORF finder were aligned using Clustal W alignment tool in the Bioedit Sequence Alignment Editor Version 7.2.5 to observe any changes in the amino acid sequences. A pairwise comparison of the retrieved amino acid sequences with the wild-type sequence was made using BLAST2 (for protein) tool at NCBI to analyze the functional equivalence of each amino acid substitutions.

#### Structure prediction of Pi-ta gene

The three-dimensional structure of *wPi-ta* protein was predicted by using Phyre2 server version 2.0 (http://www.sbg.bio.ic.ac.uk/phyre2/html/page.cgi?id=index). This server scans the annotated proteins in the SCOP and PDB databases against the query sequence and builds a model based on ten templates with the highest matches, based on heuristics to maximize confidence, percentage identity, and alignment coverage [15]. The protein structures of *Pi-ta* gene of the 47 Sri Lankan rice accessions were predicted by generating an array of plausible models and finally, the best model was selected based on the confidence percentage and the percentage of query alignment. The software HHpred 1.51, Psi-pred 2.5, Disopred 2.4, Memsat_SVM, and Poing 1.0 were used for template detection, secondary structure prediction, disorder prediction, transmembrane prediction, and multi-template modelling and ab initio, respectively.

#### Structure comparison

In the presence of significant changes among amino acids, structural differences between the *Pi-ta* protein 3D structures of each accession with *wPi-ta* protein was compared. 3D structure of proteins was compared by TM align software (https://zhanglab.ccmb.med.umich.edu/TM-align/) which is an algorithm for sequence-independent protein structure comparisons [16]. The accuracy of the alignment was determined based on the root mean square deviation (RMSD) and TM score.

### Confirmation of *in silico* analysis by Molecular marker assay

Based on the results of *in silico* analysis, 23 out of the initial 47 accessions, representing resistant and susceptible genotypes, were randomly selected for experimental confirmation by an allele-specific PCR assay (Table 4). In addition, the same assay was conducted for 25 cultivars, commonly cultivated in the Northern Province of Sri Lanka. Cultivar *Tetep* was used as a reference for the assay (Table 5).

DNA of the 23 selected accessions was extracted using PhytoSpin^TM^ Plant Genomic DNA extraction kit (Ceygen Biotech, Sri Lanka) following the manufacturer’s instructions. PCR was performed to screen the presence of disease resistant (wild type) or disease susceptible allele of *Pi-ta* gene using allele-specific primers, YL 155 (5’AGCAGGTTATAAGCTAGGCC 3’)/YL87 (5’ CTACCAACAAGTTCATCAAA 3’ for resistant allele and YL183 (5’AGCAGGTTATAAGCTAGCTAT 3’)/YL87) for susceptible allele respectively as published by Jia et al. [17]. PCR was carried out in 25 μl reactions containing; 1XPCR buffer (Promega), 2.5 mM MgCl_2_, 0.2 mM dNTP, 10 μM of each primer, 0.06 U of GoTaq DNA polymerase (Promega Corporation, USA), 2 μl of DNA. The PCR amplification was performed following the cycle; initial denaturation at 94 °C for 5 min, 35 cycles of 94 °C for 30 s, 55 °C for 45 s, 72 °C for 2 min and final extension at 72 °C for 5 min. The PCR amplicons were separated by gel electrophoresis on 1% agarose gel in 1XTAE buffer at 60 V for 1.5 h. Amplicons were visualized by staining with ethidium bromide and exposing to UV trans-illuminator (Gel documentation system, Vilber lourmat, France).

### Disease reaction (pathogenicity assay)

The seeds of the 23 selected accessions and the 25 cultivars commonly cultivated in the Northern Province of Sri Lanka (Tables 4 and 5) were surface sterilized with 70% ethyl alcohol for 2 min, rinsed in sterile distilled water, then germinated in plastic pots containing sterile soil, and placed in a greenhouse. Each pot contained 25 seeds per accession. Urea (0.2 g/pot) was applied to increase vegetative growth, and plants were watered daily. *M. oryzae* culture was incubated at room temperature (25 °C) until sporulation on a specific medium containing agar (20 g), sucrose (5 g), and water (1 l). Conidia were harvested 2 weeks after culturing, and the concentration was adjusted to 1 × 10^5^ per ml. A volume of 10 ml of the suspension (10 ml) was inoculated into each pot using an atomizer when the plants were at 4th to 5th leaf stage. After inoculation, pots were kept in a moistened chamber for 48 h and then transferred to the green house. The degree of disease on each seedling was evaluated 7 days after inoculation based on the Standard Evaluation System (SES) as described by the International Rice Research Institute (IRRI, 2013). Scores identified as 0–3 were considered to be resistant (R), 4–5 as moderately resistant (MR) and 6–9 as susceptible (S) as reported by Imam *et al*. [18]. This was followed by second scoring after a 3-day interval as reported by Shikari et al. [19].

## Results

### *In silico* analysis

Out of the 47 accessions analyzed, 37 accessions revealed 18 and 21 different single-nucleotide polymorphisms (SNPs) variations in exons 1 and 2 respectively (Supplementary tables 1 and 2), while 10 accessions (*Alagusamba*, *Honderawala*, *Pachchaiperumal*, *Podiwee*, *Pokkali*, *Race perumal*, *Samba*, *Sithaiyan kottai samba*, 3210, and BW 295-5) were identical to *wPi-ta* (*Tetep*). The detected SNPs did not lead to truncation of the ORFs. Comparison of the resulting amino acid substitutions at 27 different positions along the amino acid length is given in Table 1. Among them, 9 SNPs were noted in Nucleotide Binding Site (NBS) and 8 in LRD region resulting in 9 different allelic types (denoted as type I–IX in Table 1). The detailed amino acid variations among all the 47 accessions are given in supplementary table 3.

**Table 1:**
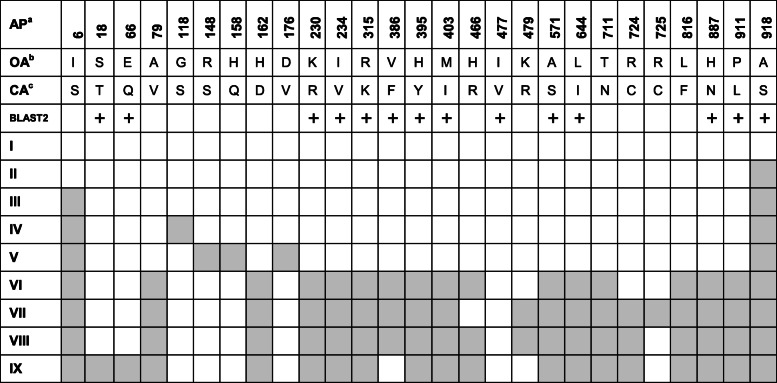
Schematic representation of positional changes along with the amino acid sequences of 47 Sri Lankan rice accessions compared to *wPi-ta* (*Tetep*). The + sign shows the functional similarities of the amino acids and the empty boxes depict the dissimilarity.

^a^Amino acid position throughout the total length of *Pi-ta* protein

^b^Original amino acid position presents in *wPi-ta* protein

^c^Changed amino acids in *Pi-ta* proteins of Sri Lankan rice accessions

Allelic types are numbered as I–IX (Accessions, as per the supplementary table 4, belonging to Type I—1,9,25,29,30,33,35,39,44,46; Type II—17, 42; Type III—3,4,6,7,11,12,14,15,16,20,21,23,24,26, 37,40,41,47; Type IV—2,8,28,31,32,36,43; Type V—5; Type VI—38; Type VII—22; Type VIII—10, 13, 27, 34,19; Type IX—45), where the type I is the wild type (*wPi-ta*); the positions at which the amino acid substitutions were observed are highlighted in grey for each allelic type.

The maximum number (*n* = 19) of amino acid polymorphisms (APs) was noted in the six accessions: *Hodarawala*, *Karuthaheenati*, *Mathalowa*, *Podiheenati*, *Ranruwan,* and A69.1. These accessions shared 97.95% sequence identity with *wPi-ta*, in blast2 analysis followed by *Mudaligawee* and *Sayam* which revealed 18 and 17 Aps, respectively with 98.06% sequence identity. The accessions *Kuruluwee* white and *Wir*1391 revealed AP in the 918th amino acid position alone. All the 37 accessions displayed alanine to serine substitution at the 918th position, and this was described as a functional mutation determining resistance by altering the binding specificity [5] (Table 2). This mutation is found in the LRD region, directly involving with the pathogen recognition.

**Table 2:** Comparison of *Pi-ta* protein sequences of 47 Sri Lankan rice accessions with *wPi-ta* (*Tetep*, GenBank accession number GQ918486.1) using Clustal W, Bioedit 7.2.5. Highlighted in black are the amino acids that contributed to the resistant phenotype due to the absence of the functional mutation at the 918th amino acid position

No	Accession name	IRGC No	
0	*w*Pita		QGLLSFFLSL PWLLSLPAMH LQPDLMIV
1	Alagusamba	IRGC 8944-2	QGLLSFFLSL PWLLSLPMH LQPDLMIV
2	Balasuriya	IRGC 66509-1	QGLLSFFLSL PWLLSLPSMH LQPDLMIV
3	Chandina	IRGC 36420-1	QGLLSFFLSL PWLLSLPSMH LQPDLMIV
4	Galawaka handeran	IRGC 31381-1	QGLLSFFLSL PWLLSLPSMH LQPDLMIV
5	Godawel	IRGC 15750	QGLLSFFLSL PWLLSLPSMH LQPDLMIV
6	Halsuduheenati	IRGC 15599-1	QGLLSFFLSL PWLLSLPSMH LQPDLMIV
7	Heendikwee	IRGC 15587-2	QGLLSFFLSL PWLLSLPSMH LQPDLMIV
8	Herath Banda	IRGC 67630-1	QGLLSFFLSL PWLLSLPSMH LQPDLMIV
9	Honderawala	IRGC 47372-1	QGLLSFFLSL PWLLSLPMH LQPDLMIV
10	Hodarawala	IRGC 67631-1	QGLLSFFLSL LWLLSLPSMH LQPDLMIV
11	Kahatawee	IRGC 12004-1	QGLLSFFLSL PWLLSLPSMH LQPDLMIV
12	Kalu Ilankayan	IRGC 36270-1	QGLLSFFLSL PWLLSLPSMH LQPDLMIV
13	Karutha seenati	IRGC 15515-2	QGLLSFFLSL PWLLSLPSMH LQPDLMIV
14	Kotteyaran	IRGC 47383-1	QGLLSFFLSL PWLLSLPSMH LQPDLMIV
15	Kula karupan	IRGC 55328-1	QGLLSFFLSL PWLLSLPSMH LQPDLMIV
16	Kurkaruppan	IRGC 15449-1	QGLLSFFLSL PWLLSLPSMH LQPDLMIV
17	Kurulu wee (White)	IRGC 66518-1	QGLLSFFLSL PWLLSLPSMH LQPDLMIV
18	Kurulutudu	IRGC 36304-1	QGLLSFFLSL PWLLSLPSMH LQPDLMIV
19	Matholuwa	IRGC 8901-1	QGLLSFFLSL PWLLSLPSMH LQPDLMIV
20	Moddai karupan	IRGC 15465-1	QGLLSFFLSL PWLLSLPSMH LQPDLMIV
21	Murunga	IRGC 15428-1	QGLLSFFLSL PWLLSLPSMH LQPDLMIV
22	Mudalige wee	IRGC 74706-1	QGLLSFFLSL PWLLSLPSMH LQPDLMIV
23	Muttu Samba	IRGC 36333-1	QGLLSFFLSL PWLLSLPSMH LQPDLMIV
24	Nalumoolaikarupan	IRGC 8993-1	QGLLSFFLSL PWLLSLPSMH LQPDLMIV
25	Pachchaperumal	IRGC 3474-1	QGLLSFFLSL PWLLSLPMH LQPDLMIV
26	Periya vellai	IRGC 15475-1	QGLLSFFLSL PWLLSLPSMH LQPDLMIV
27	Podi heenati	IRGC 36345-1	QGLLSFFLSL PWLLSLPSMH LQPDLMIV
28	Pannithi	IRGC 51049-1	QGLLSFFLSL PWLLSLPSMH LQPDLMIV
29	Podiwee	IRGC 11938-1	QGLLSFFLSL PWLLSLPMH LQPDLMIV
30	Pokkali	IRGC 8948-1	QGLLSFFLSL PWLLSLPMH LQPDLMIV
31	Puttu nellu	IRGC 55346-1	QGLLSFFLSL PWLLSLPSMH LQPDLMIV
32	Rangoon samba	IRGC 11940-1	QGLLSFFLSL PWLLSLPSMH LQPDLMIV
33	Race perumal	IRGC 55347-1	QGLLSFFLSL PWLLSLPMH LQPDLMIV
34	Ranruwan	IRGC 36360-1	QGLLSFFLSL PWLLSLPSMH LQPDLMIV
35	Samba	IRGC 11993-1	QGLLSFFLSL PWLLSLPMH LQPDLMIV
36	Sinna sithira kalli	IRGC 51064-1	QGLLSFFLSL PWLLSLPSMH LQPDLMIV
37	Sigardis	IRGC 15555-1	QGLLSFFLSL PWLLSLPSMH LQPDLMIV
38	Sayam	IRGC 31538-1	QGLLSFFLSL PWLLSLPSMH LQPDLMIV
39	Sithaiyankottai samba	IRGC 50155-1	QGLLSFFLSL PWLLSLPMH LQPDLMIV
40	Sudu karayal	IRGC 15348-1	QGLLSFFLSL PWLLSLPSMH LQPDLMIV
41	2Vellai kollumban	IRGC 15517-1	QGLLSFFLSL PWLLSLPSMH LQPDLMIV
42	WIR 1391	IRGC 51605-1	QGLLSFFLSL PWLLSLPSMH LQPDLMIV
43	105	IRGC 40896-1	QGLLSFFLSL PWLLSLPSMH LQPDLMIV
44	3210	IRGC 1116950-1	QGLLSFFLSL PWLLSLPMH LQPDLMIV
45	A69-1	IRGC 55305-1	QGLLSFFLSL LWLLSLPSMH LQPDLMIV
46	Bw295-5	IRGC 63098-1	QGLLSFFLSL PWLLSLPMH LQPDLMIV
47	H6	IRGC 157-1	QGLLSFFLSL LWLLSLPSMH LQPDLMIV

**Fig. 1:**
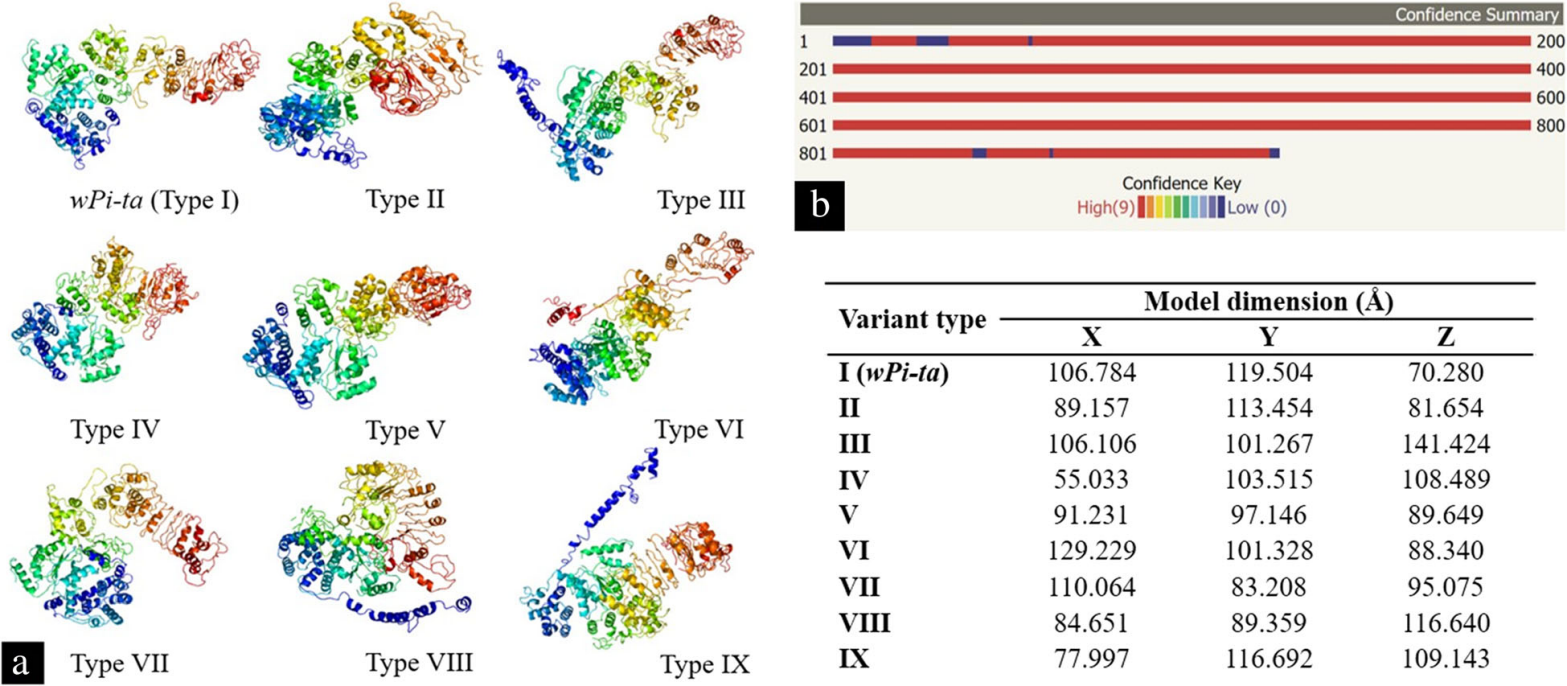
Details of structure prediction. **a** Predicted models of *Pi-ta* protein variants and *wPi-ta* (Type I) using the Phyre2 server. The images are coloured by rainbow N to C terminus. **b** Confidence summary of predicted models, and the table has shown the dimension of predicted models of *Pi-ta* protein variants using three coordinates (X-axis, Y-axis, Z-axis), in Angstrom (Å)

There were 8 APs noted in the LRD region including one at position 918. Four positions (711, 724, 725, and 816) had nonequivalent amino acid substitutions. Interestingly, alanine to serine substitution left a plus (+) sign, revealing functional equivalence of the amino acids even though this position confers recognition specificity. Nine templates with the confidence percentage of > 90 (PDB codes: c4kxfP, c3qflA, c3iz8C, c3iz8A, c1vt4K, c1vt4N, c3iz8G, c4ecnA, and c2a5yB) were selected to model the protein based on heuristics to maximize confidence, percentage identity, and alignment coverage in Phyre 2. Considering the unavailability of resolved 3D structure in the database, structural prediction was carried out for further analysis. Structures of 47 *Pi-ta* genes and *wPi-ta* of the templates (PDB code)—c2a5yB, c1vt4K, c3iz8C, c3iz8A, c3iz8G, c1vt4N, c3iz8E, c3iz8B, c1vt4P, c1vt4O, c1vt4L, c3iz8D, c3iz8F, c1vt4M, c3iz8H, c1vt4J, c1z6tC, c4kxfP, c5juyB, c1vt4I—were derived with 100% confidence level, based on homology modelling. Ninety-five percent of the amino acids were modelled at > 90% confidence level, where it is assumed that the modelled protein adopts the overall fold shown and that the core of the protein is modelled at high accuracy, while 47 residues were modelled by *ab initio* modelling. The 3D structures of the nine allelic types (Types I to IX: Table 1) revealed amino acid variations leading to alterations in the protein structure resulting in different structural dimensions (Fig. 1).

**Fig. 2:**
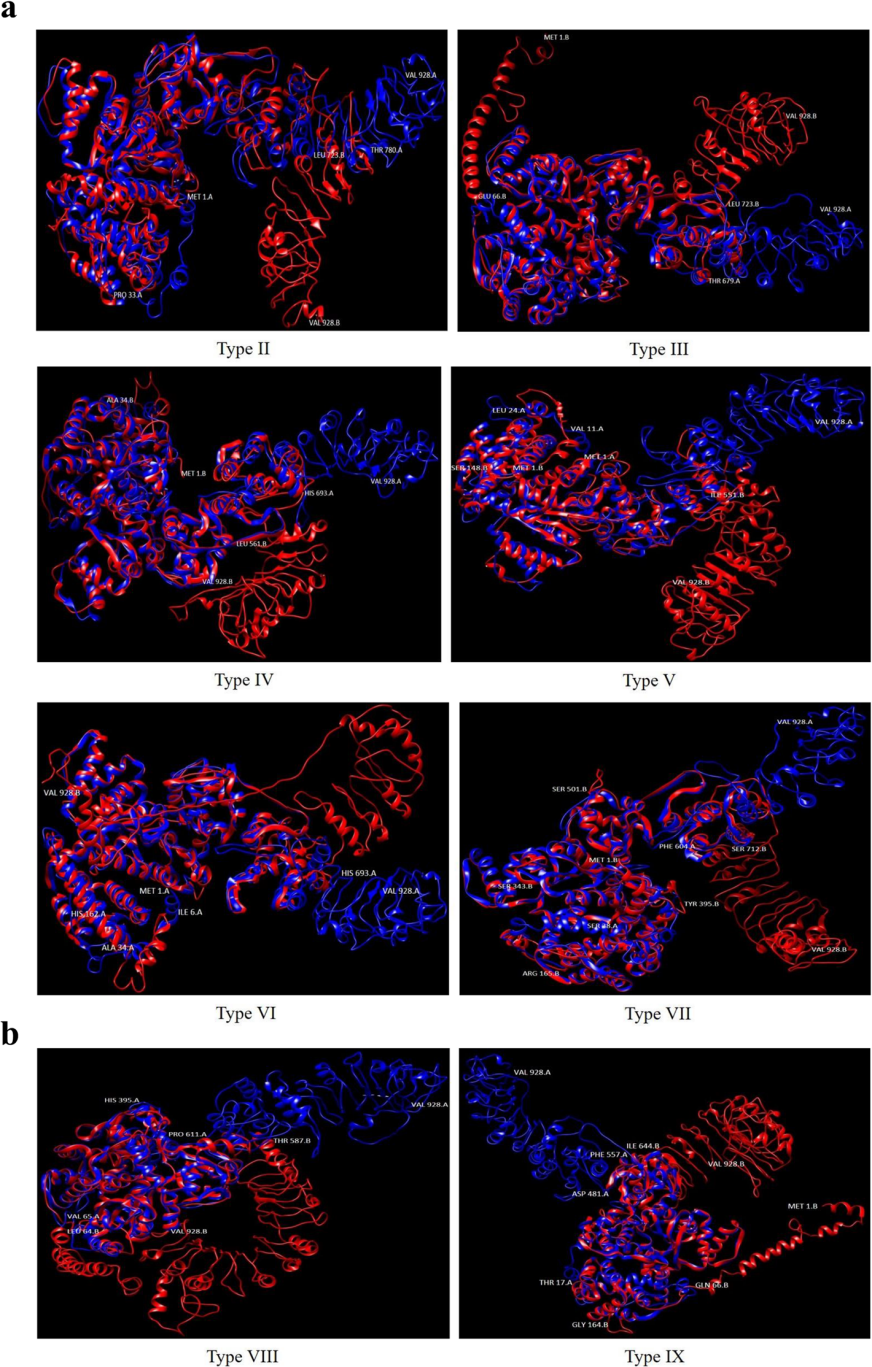
**a** Superimposition of *wPi-ta* with the other eight identified variant allelic types (Types II–VII) in TM-align software. **b** Superimposition of *wPi-ta* with the other eight identified variant allelic types (Types VIII and IX) in TM-align software

Structural superimposition of the nine allelic types (Table 1) is presented in Fig. 2a, b.

The results from the superimposition analysis clearly indicated that the variation in amino acid length, resulting in higher Root Mean Square Deviation (RMSD) values (Table 3) even where a single AP is detected. TM score for all the superimpositions were above 0.5 and below 1.0, which assumes that the structures are roughly the same in folds in both SCOP (fold) and CATH (topology) structural classification databases.

**Table 3:** The values of root mean square deviation (RMSD) and TM score for superimposed *Pi-ta* protein variants with *wPi-ta*

*Pi-ta* variant	RMSD	TM score
II	4.32	0.67242
III	3.06	0.63381
IV	3.48	0.67963
V	4.95	0.57160
VI	3.56	0.67246
VII	2.48	0.64665
VIII	4.03	0.53159
IX	2.63	0.51643

### Molecular marker assay

**Fig. 3:**
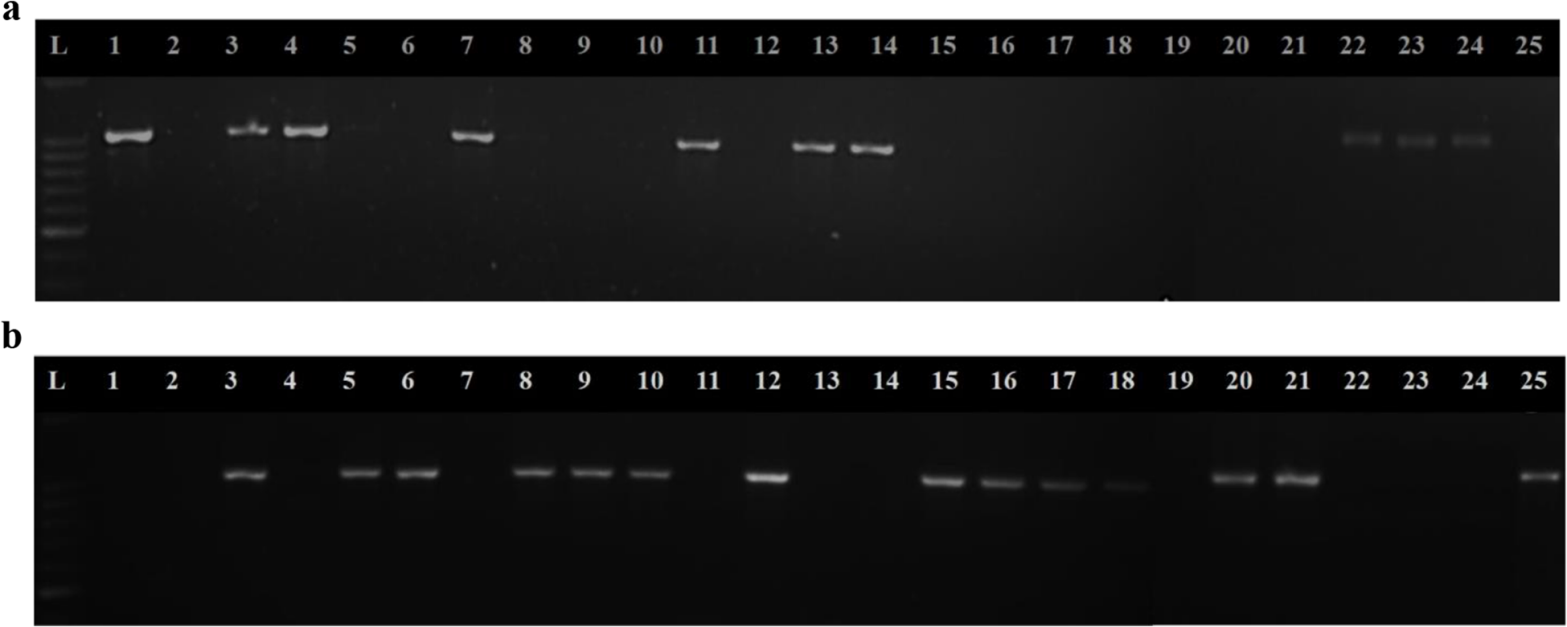
**a** The presence or absence of resistant *wPi-ta* allele using the specific marker YL155/YL87. L: 100 bp ladder, Lane 1: Positive control (*Tetep*), Lane 2: Negative control, Lane 3: *Honderawala*, Lane 4: *Podiwee*, Lane 5: *Podiheenati*, Lane 6: *Periyavellai*, Lane 7: Bw 295-5, Lane 8: *Sinna sithirakalli*, Lane 9: A 69-1, Lane 10: *Ranruwan*, Lane 11: *Pokkali*, Lane 12: *Mudaligawee*, Lane 13: *Pachchaperumal*, Lane 14: *Race perumal*, Lane 15: *Godawel*, Lane 16: *Karuthaheenati*, Lane 17: *Vellaikolumban*, Lane 18: 105, Lane 19: *KaluIlangayan*, Lane 20: H6, Lane 21: *Sigardis*, Lane 22: *Samba*, Lane 23: *Alagusamba*, Lane 24: *Sithayakottai samba*, Lane 25: *Kahatawee.* ***b*** The presence of the 918th mutation in *Pi-ta* gene using the specific marker YL183/YL87. L: 100 bp ladder, Lane 1: Positive control (*Tetep*), Lane 2: Negative control, Lane 3: *Podiheenati*, Lane 4: *Podiwee*, Lane 5: *KaluIlangayan*, Lane 6: *Periyavellai*, Lane 7: Bw 295-5, Lane 8: *Sinna sithirakalli*, Lane 9: A 69-1, Lane 10: *Ranruwan*, Lane 11: *Pokkali,* Lane 12: Sigardis, Lane 13: *Pachchaperumal,* Lane 14: *Race perumal*, Lane 15: *Godawel*, Lane 16: *Karuthaheenati*, Lane 17: *Vellaikolumban*, Lane 18: 105, Lane 19: *Honderawala*, Lane 20: H6, Lane 21: *Kahatawee,* Lane 22: *Samba*, Lane 23: *Alagusamba*, Lane 24: *Sithayankottai samba*, Lane 25: *Mudaligawee*

The gel image in Fig. 3a shows the presence of *Pi-ta* resistant gene in 9 rice accessions including *Tetep* (*wPi-ta*). Nine accessions produced amplicons (1042 bp) for the resistant SSR loci YL155/YL87 (Fig. 3a) and 14 accessions which did not produce the amplicons for the above loci, produced amplicons for the susceptible allele; YL183/YL87, at the expected size of 1043 bp (Fig. 3b). The results were consistent with *in silico* analysis as summarized in Table 4.

**Table 4:** Overview of the results for the assays conducted to evaluate the presence of *Pi-ta* gene in Sri Lankan rice accessions (PGRC—Plant Genetic Resources Institute of Sri Lanka; *R* resistant, *S* susceptible; ‘+’ represents the presence and ‘-’ represents the absence of amplicons in the specific PCR assays).

Accession name	Accession no.	*In silico*	Severity scale	Disease reaction status	YL155/YL87	YL183/YL87
*Alagusamba*	IRGC 8944-2	R	0	R	+	-
*Podiwee*	IRGC 11938-1	R	1	R	+	-
H6	IRGC 157-1	S	5	MR	-	+
*Race perumal*	IRGC 55347-1	R	6	S	+	-
BW 295-5	IRGC 63098-1	R	0	R	+	-
*Samba*	IRGC 11993-1	R	0	R	+	-
*Pachchaiperumal*	IRGC 3474-1	R	1	R	+	-
*Honderawala*	IRGC 67631-1	R	3	R	+	-
*Pokkali*	IRGC 8948-1	R	2	R	+	-
A69-1	IRGC 55305-1	S	5	MR	-	+
*Periyavellai*	IRGC 15475-1	S	6	S	-	+
*Kahatawee*	IRGC 12004-1	S	7	S	-	+
*Karuthaheenati*	IRGC 15515-2	S	0	R	-	+
*Sigardis*	IRGC 15555-1	S	4	MR	-	+
105	IRGC 40896-1	S	5	MR	-	+
*Godawel*	IRGC 15750-1	S	5	MR	-	+
*Sinna sithirai kalli*	IRGC 51064-1	S	4	MR	-	+
*Vellaikolumban*	IRGC 15517-1	S	5	MR	-	+
*Podiheenati*	IRGC 36345-1	S	4	MR	-	+
*Mudaligawee*	IRGC 74706-1	S	7	S	-	+
*Kaluilangayan*	IRGC 36270-1	S	6	S	-	+
*Ranruwan*	IRGC 36360-1	S	5	MR	-	+
*Sithayankottai samba*	IRGC 50155-1	R	2	R	+	-

Among the 25 tested cultivars from the Northern Province of Sri Lanka, *Attakkari*, Bw 372, *Moddaikaruppan*, *Suwandal*, At 402, Bg 366, Bg 450, and *Karuththaheenati* did not amplify the resistant SSR loci; YL155/YL87 (Supplementary figure 1).

### Pathogenicity assay

**Fig. 4:**
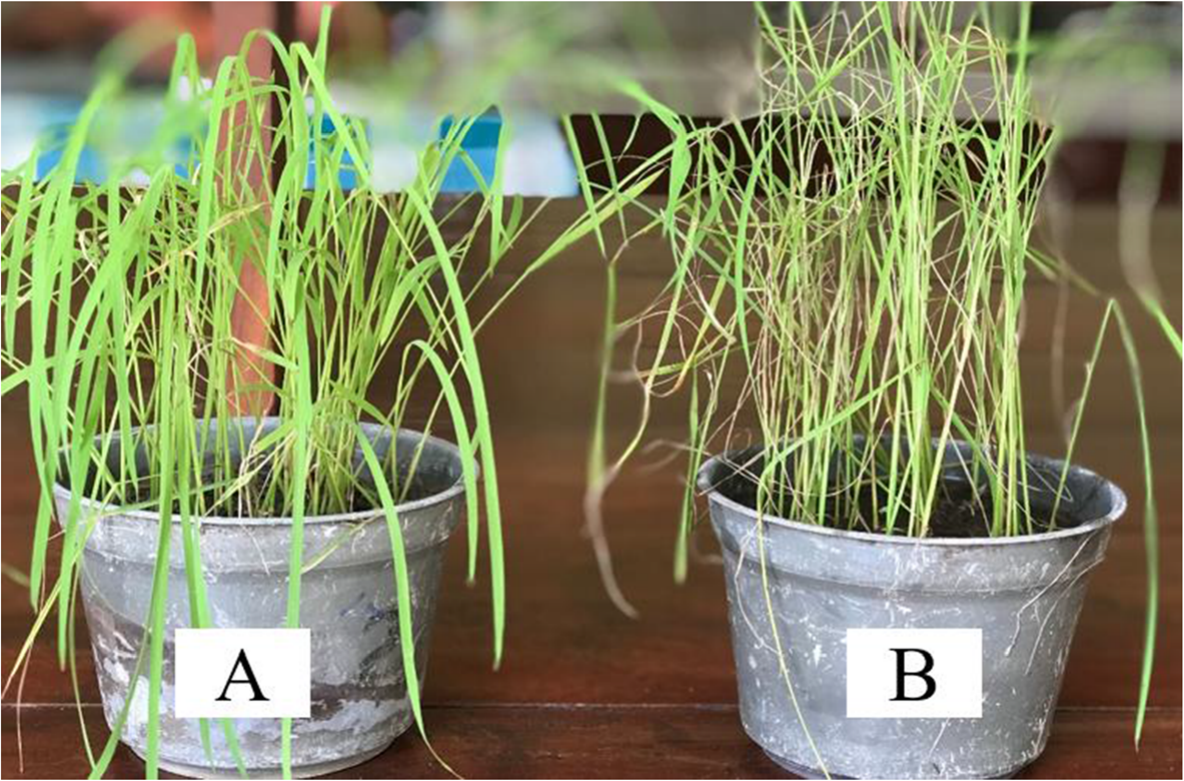
Seedlings of rice cultivars after 14 days of inoculation of *M. oryzae* conidia. Bg 360 (**A**) and *Attakari* (**B**) recorded severity scales 1 and 6 respectively

This assay revealed that among the nine resistant accessions identified in the molecular marker assay, eight were consistent with the results of *in-silico* analysis except for *Race perumal* while *Karuthaheenati* showed resistant reaction out of 14 accessions revealed susceptibility (Table 4). Pathogenicity assay was performed for these two accessions again to validate the results. The same results were obtained in the pathogenicity assay as well. Among the tested cultivars from the Northern Province, 13 scored resistant (R) phenotype, 11 moderately resistant (MR), and one susceptible (S) response. Figure 4 illustrates the two cultivars with the minimum (1 for Bg360) and maximum (6 for *Attakari*) severity scales observed in the pathogenicity assay performed.

Among the eight cultivars which did not amplify the resistant *wPi-ta* allele, the cultivar *Attakari* revealed S response in the pathogenicity assay, while cultivars *Moddaikaruppan* and *Suwandal* recorded R response and the remaining five cultivars (Bw372, At402, *Karuthaheenati,* Bg366, and Bg450) showed MR response (Table 5).

**Table 5:** List of 25 cultivars from the Northern Province of Sri Lanka and their response in the pathogenicity assay (*KN*, Kilinochchi; *MU*, Mullaitheevu; *MA*, Mannar; *VA*, Vavuniya; *JFN*, Jaffna; ‘+’ represents the presence and ‘-’ represents the absence of amplicon in the specific PCR assays)

Cultivar	District	Disease symptom		YL155/ YL87
		Severity scale	Status	
Bg 360	KN, MU, VA, MA, JAF	1	R	+
At 362	KN, MU, VA	1	R	+
Bg 358	KN, MU, VA, MA	3	R	+
Bg 406	KN, VA, MA	5	MR	+
At 308	KN, MU, VA, MA	2	R	+
Bg 300	KN, MU, VA, MA, JAF	5	MR	+
*Attakari*	KN, MU, VA	6	S	-
*Moddaikaruppan*	KN, MU, JAF	0	R	-
*Suwandal*	KN, VA	1	R	-
Bw 372	KN	5	MR	-
Bw 367	MU, VA, MA	1	R	+
At 353	MU	2	R	+
Bg 250	MU, MA	4	MR	+
At 402	MU	2	MR	-
*Pachchaiperumal*	MU, JAF	2	R	+
*Karuthaheenati*	MU	0	MR	-
Bg 352	VA, MA	2	R	+
Bg 366	VA	1	MR	-
Bg 450	VA, MA	0	MR	-
Ld 365	MA	2	R	+
Co10	KN, JAF, MU	5	MR	+
Bg 369	KN. MU	1	R	+
Bw 351	KN	4	MR	+
Bg 251	KN, VA, MA	5	MR	+
Bg 94-1	MU, MA	2	R	+

## Discussion

Resistance genes offer the most effective and environmentally safe option for the management of the pathogen [20]. *Pi-ta* is a well-studied blast resistant gene [21–23]. Huang et al. [24] studied the molecular evolution of *Pi-ta* gene in wild rice *O. rufipogon* and identified two haplogroups, H1 and H2, with the amino acid Ala-918 present in H1 of the LRR domain of *Pi-ta* gene displaying a close relationship with the resistant phenotype. Yan et al. [25] analyzed the *Pi-ta* gene diversity and reported 78 polymorphic nucleotide sites which leading to 22 amino acid variations with mutations reported at the 148th, 158th, 176th, and 641st amino acid positions failing to have an impact on the resistance phenotype. Among the *Pi-ta* variants reported by Wang et al. [4], 105 polymorphic sites were identified with 27 sites resulting in amino acid substitutions, where the higher number of polymorphism was observed in intron regions rather than in exons. The results also suggested that alanine at amino acid position 918 of LRD is critical for the integrity of the *Pi-ta* protein, and isoleucine at position 6 could result in an insignificant non synonymous change of the *Pi-ta* protein. A sequence analysis of 1790 accessions was conducted by Wang et al. [26] to characterize *Pi-ta*, and *Pi-ta–*independent resistance genes and revealed functional polymorphism at the base position 918 to exhibit resistant phenotype in the pathogenicity assay. Exploitation of valuable sources to obtain resistant *Pi-ta* gene in local germplasm is an essential yet a challenging task of rice breeders. Identification of major rice blast resistant genes in local elite cultivars improves the value of germplasm sources in rice breeding programs. The current study revealed the *Pi-ta* gene diversity of the *indica* rice accessions and cultivars for the exploitation in breeding and management of rice blast disease.

The results of *in silico* analysis from the current study revealed that ten among the 47 accessions tested to be identical to *wPi-ta* gene of variety *Tetep*. Coding nucleotide sequence polymorphism of 47 accessions was compared by Clustal W multiple alignment. The pairwise alignment revealed 10 accessions consist of identical sequences with the *wPi-ta* gene, while the rest of the accessions displayed nucleotide polymorphisms resulting to nucleotide substitutions leading to 27 different amino acid substitutions in the studied accessions. Mutations in the LRD region is highly significant in pathogen recognition [27], and eight amino acid polymorphisms were noted in the LRD including four nonequivalent amino acid substitutions. Alanine to serine substitution plays a major role in defense response where *Pi-ta* protein confers recognition specificity with *Avr-Pita* being an elicitor for hypersensitive reactions [17, 28]. In this study, 37 accessions were revealed to have this mutation in the LRD region.

The Phyre2 server produced a set of potential 3D models of *Pi-ta* protein based on alignment to known protein structures in the PDB database. The pipeline involved detecting sequence homologues with PSI-Blast; predicting secondary structure and disorder with Psi-pred and Diso-pred; constructing a hidden Markov model (HMM) of the sequence based on the homologues detected; constructing 3D models of the protein based on the alignments between the HMM of the sequence and the HMMs of known structures; modelling insertions and deletions using a loop library, a fitting procedure (cyclic coordinate descent), and a set of empirical energy terms; modelling of amino acid side chains using a rotamer library from Roland Dunbrack’s laboratory; and the server’s own implementation of a fast graph-based approach (R3) to optimize the choice of rotamer for each side chain while trying to avoid steric clashes, the top model (if sufficiently confident) submitted for binding site prediction by 3DLigandSite, transmembrane helix, and topology prediction by memsat-svm. The predicted structures of the studied allelic variants (Types II–IX) varied significantly from the wild type (Type I), even with a single mutation (for the type II) at the amino acid position 918. The impact of this specific mutation at amino acid position 918 has been reported previously [2]. However, in the current study, it has been shown that the mutations at the other positions also contributed to structural changes. This was evident in two types, namely Types XIII and IX, where mutations were observed in 19 positions. All except two mutations were observed at the same positions in both types. Even though the variations between these two types are very low, both exhibited significant structural variations.

RMSD scores, a measure of accuracy when comparing different models from a particular dataset, from the structural superimposition were consistent with the number of mutations and confirmed the mutation effect on the structure of the resulting protein. Since, the LRD region was involved with the binding to *Avr* protein of the pathogen, substitutions in the region may make differences in the binding ability [29].

The allele-specific YL155/YL87 and YL183/YL87 primer pairs were selected specifically to differentiate resistant and susceptible genotypes, respectively by amplifying the region of functional gene and mutation at the 918th amino acid position with the same reverse primer. These results were consistent with the *in silico* analysis facilitating the use of findings to detect the resistant *Pi-ta* gene in rice cultivars as a validated method. The pathogenicity assay revealed that out of 14 susceptible accessions revealed from the *in silico* and molecular marker assays, four with the severity scale of 6 and above (*Periyavellai*, *Kahatawee*, *Mudaligawee*, and *KaluIlangayan*) were highly infected with blast, while the remaining ten were moderately resistant. These results indicated that even though they were susceptible with respect to *Pi-ta* gene in the *in silico* analysis, the degree of resistance might vary slightly, due to the influence of other blast resistant genes. But those susceptible accessions amplified the resistant allele for YL183/YL8. Similar results were observed by Jayawardana et al. [30] where some resistant varieties (Bg 300, Bg 348, Ptb33) lack the functional *Pi-ta* (*wPi-ta*) gene. The results derived from the *in silico* analysis were consistent with the resistant phenotype and DNA marker-based molecular screening. As a result, these accessions identified to be resistant can be used as a source of *Pi-ta* gene for future gene pyramiding work for rice blast resistance. The results of the validation trial with local germplasm clearly indicated the resistant response of the cultivars possessing the *wPi-ta* allele, while the study further revealed the presence of other genes that might be responsible for resistance in cultivars which did not contain the *Pi-ta* gene.

## Conclusions

*In silico* analysis of sequence variations of the *Pi-ta* gene in Sri Lankan rice varieties revealed that some cultivars contain resistant *Pi-ta* alleles as similar to that of *Tetep,* the donor rice variety of the *Pi-ta* gene. Protein modelling revealed the lack of truncation in the amino acid sequence of the *Pi-ta* protein but revealed variations in the amino acid sequence, especially in the LRD region, resulting from the single-nucleotide polymorphisms of the *Pi-ta* gene. Molecular marker assay in detecting the resistant and susceptible *Pi-ta* alleles and the pathogenicity assay confirmed the validity of the information derived from the *in silico* analysis. The ten accessions with resistant *Pi-ta* alleles identified in the present study would be useful genetic resources for future breeding programmes. The involvement of genes other than *Pi-ta* in blast resistance should also be studied. A detailed research on such sources would also be extremely useful in breeding rice for resistance to blast disease.

## Supplementary Information

Additional file 1: **Supplementary figure 1.** The presence of resistant *wPi-ta* allele using the specific marker YL155/YL87. L: 100bp ladder, Lane 1: Positive control (*Tetep*), Lane 2: Negative control, Lane 3: Bg 360, Lane 4: At 362, Lane 5: *Attakari*,, Lane 6: *Moddaikaruppan,* Lane 7: Bg 358, Lane 8: *Suwandal*, Lane 9: Bw 372, Lane 10: Bg 406*,* Lane 11: *Pachchaiperumal*, Lane 12: At 308, Lane 13: Bg 300, Lane 14: At 402, Lane 15: Karuthaheenati, Lane 16: Ld 365, Lane 17: Bg 366, Lane 18: Bg 94-1, Lane 19: Bg 251, Lane 20: Co10, Lane 21: Bg 369, Lane 22: Bw 351, Lane 23: Bg 352, Lane 24: Bg 250, Lane 25: Bw 367, Lane 26: At 353*,* Lane 27: Bg 450

Additional file 2: **Supplementary table 1**. The presence of mutation in the exon 1 of Pi-ta gene in 47 Sri Lankan rice accessions

Additional file 3: **Supplementary table 2**. The presence of mutation in the exon 2 of Pi-ta gene in 47 Sri Lankan rice accessions

Additional file 4: **Supplementary Table 3**. Summary of mutation in the Pi-ta protein of 47 Sri Lankan rice accessions

Additional file 5: **Supplementary table 4**. List of 47 Sri Lankan rice accessions deposited in Rice SNP-Seek Database of the International Rice Research Institute

## Data Availability

Not applicable
