# Supplementary material for: In silico molecular and morphological analysis of rice blast resistant gene Pi-ta in Sri Lankan rice germplasm
Source: J Genet Eng Biotechnol. 2021 Oct 21;19:163. doi: 10.1186/s43141-021-00239-7 (PMC8531186; doi:10.1186/s43141-021-00239-7)
Supplement: Supplementary file 1 — Supplementary figure 1. The presence of resistant wPi-ta allele using the specific marker YL155/YL87. L: 100bp ladder, Lane 1: Positive control (Tetep), Lane 2: Negative control, Lane 3: Bg 360, Lane 4: At 362, Lane 5: Attakari,, Lane 6: Moddaikaruppan, Lane 7: Bg 358, Lane 8: Suwandal, Lane 9: Bw 372, Lane 10: Bg 406, Lane 11: Pachchaiperumal, Lane 12: At 308, Lane 13: Bg 300, Lane 14: At 402, Lane 15: Karuthaheenati, Lane 16: Ld 365, Lane 17: Bg 366, Lane 18: Bg 94-1, Lane 19: Bg 251, Lane 20: Co10, Lane 21: Bg 369, Lane 22: Bw 351, Lane 23: Bg 352, Lane 24: Bg 250, Lane 25: Bw 367, Lane 26: At 353, Lane 27: Bg 450 [file 43141_2021_239_MOESM1_ESM.docx]

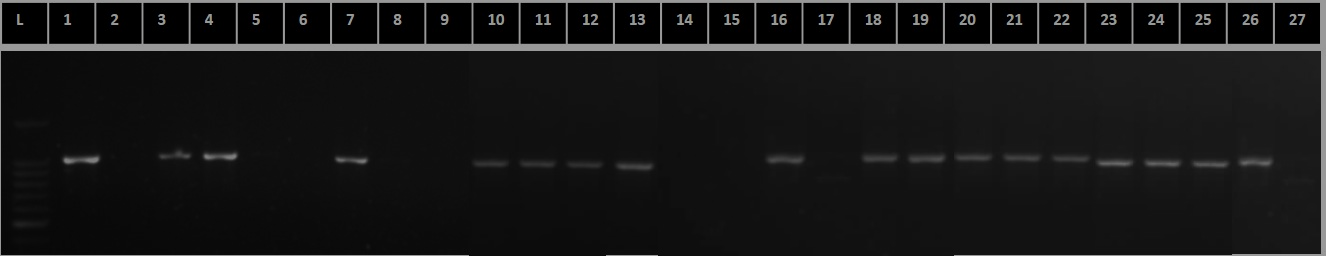


**Supplementary figure 1:** The presence of resistant *wPi-ta* allele using the specific marker YL155/YL87. L: 100bp ladder, Lane 1: Positive control (*Tetep*), Lane 2: Negative control, Lane 3: Bg 360, Lane 4: At 362, Lane 5: *Attakari*,, Lane 6: *Moddaikaruppan,* Lane 7: Bg 358, Lane 8: *Suwandal*, Lane 9: Bw 372, Lane 10: Bg 406*,* Lane 11: *Pachchaiperumal*, Lane 12: At 308, Lane 13: Bg 300, Lane 14: At 402, Lane 15: Karuthaheenati, Lane 16: Ld 365, Lane 17: Bg 366, Lane 18: Bg 94-1, Lane 19: Bg 251, Lane 20: Co10, Lane 21: Bg 369, Lane 22: Bw 351, Lane 23: Bg 352, Lane 24: Bg 250, Lane 25: Bw 367, Lane 26: At 353*,* Lane 27: Bg 450
