# Supplementary material for: In silico molecular and morphological analysis of rice blast resistant gene Pi-ta in Sri Lankan rice germplasm
Source: J Genet Eng Biotechnol. 2021 Oct 21;19:163. doi: 10.1186/s43141-021-00239-7 (PMC8531186; doi:10.1186/s43141-021-00239-7)
Supplement: Supplementary file 2 — Supplementary table 1. The presence of mutation in the exon 1 of Pi-ta gene in 47 Sri Lankan rice accessions [file 43141_2021_239_MOESM2_ESM.docx]

Supplementary table 1: The presence of mutation in the exon 1 of Pi-ta gene in 47 Sri Lankan rice accessions

| **No** | **Name** | **Accession No** | **1** | **138** | **245** | **256** | **418** | **461** | **471** | **501** | **536** | **555** | **561** | **593** | **663** | **696** | **709** | **749** | **892** | **928** |
| --- | --- | --- | --- | --- | --- | --- | --- | --- | --- | --- | --- | --- | --- | --- | --- | --- | --- | --- | --- | --- |
|  | Alagusamba | IRGC 8944-2 |  |  |  |  |  |  |  |  |  |  |  |  |  |  |  |  |  |  |
|  | Balasuriya | IRGC 66509-1 |  |  |  |  |  |  |  |  |  |  |  |  |  |  |  |  |  |  |
|  | Chandina | IRGC 36420-1 |  |  |  |  |  |  |  |  |  |  |  |  |  |  |  |  |  |  |
|  | Galawaka handeran | IRGC 31381-1 |  |  |  |  |  |  |  |  |  |  |  |  |  |  |  |  |  |  |
|  | Godawel | IRGC 15750-1 |  |  |  |  |  |  |  |  |  |  |  |  |  |  |  |  |  |  |
|  | Halsuduheenati | IRGC 15599-1 |  |  |  |  |  |  |  |  |  |  |  |  |  |  |  |  |  |  |
|  | Heendikwee | IRGC 15587-2 |  |  |  |  |  |  |  |  |  |  |  |  |  |  |  |  |  |  |
|  | Herath Banda | IRGC 67630-1 |  |  |  |  |  |  |  |  |  |  |  |  |  |  |  |  |  |  |
|  | Honderawala | IRGC 47372-1 |  |  |  |  |  |  |  |  |  |  |  |  |  |  |  |  |  |  |
|  | Hodarawala | IRGC 67631-1 |  |  |  |  |  |  |  |  |  |  |  |  |  |  |  |  |  |  |
|  | Kahatawee | IRGC 12004-1 |  |  |  |  |  |  |  |  |  |  |  |  |  |  |  |  |  |  |
|  | Kalu Ilankalayan | IRGC 36270-1 |  |  |  |  |  |  |  |  |  |  |  |  |  |  |  |  |  |  |
|  | Karutha Seenati | IRGC 15515-2 |  |  |  |  |  |  |  |  |  |  |  |  |  |  |  |  |  |  |
|  | Kotteyaran | IRGC 47383-1 |  |  |  |  |  |  |  |  |  |  |  |  |  |  |  |  |  |  |
|  | Kula karuppan | IRGC 55328-1 |  |  |  |  |  |  |  |  |  |  |  |  |  |  |  |  |  |  |
|  | Kurkaruppan | IRGC 15449-1 |  |  |  |  |  |  |  |  |  |  |  |  |  |  |  |  |  |  |
|  | Kurulu wee (white) | IRGC 66518-1 |  |  |  |  |  |  |  |  |  |  |  |  |  |  |  |  |  |  |
|  | Kurulutudu | IRGC 36304-1 |  |  |  |  |  |  |  |  |  |  |  |  |  |  |  |  |  |  |
|  | Matholuwa | IRGC 8901-1 |  |  |  |  |  |  |  |  |  |  |  |  |  |  |  |  |  |  |
|  | Moddai karuppan | IRGC 15465-1 |  |  |  |  |  |  |  |  |  |  |  |  |  |  |  |  |  |  |
|  | Murunga | IRGC15428-1 |  |  |  |  |  |  |  |  |  |  |  |  |  |  |  |  |  |  |
|  | Mudalige wee | IRGC 74706-1 |  |  |  |  |  |  |  |  |  |  |  |  |  |  |  |  |  |  |
|  | Muttu Samba | IRGC 36333-1 |  |  |  |  |  |  |  |  |  |  |  |  |  |  |  |  |  |  |
|  | Nalumoolai Karuppan | IRGC 8993-1 |  |  |  |  |  |  |  |  |  |  |  |  |  |  |  |  |  |  |
|  | Pachchaperumal | IRGC 3474-1 |  |  |  |  |  |  |  |  |  |  |  |  |  |  |  |  |  |  |
|  | Periya Vellai | IRGC 15475-1 |  |  |  |  |  |  |  |  |  |  |  |  |  |  |  |  |  |  |
|  | Podi heenati | IRGC 36345-1 |  |  |  |  |  |  |  |  |  |  |  |  |  |  |  |  |  |  |
|  | Pannithi | IRGC 51049-1 |  |  |  |  |  |  |  |  |  |  |  |  |  |  |  |  |  |  |
|  | Podiwee | IRGC 11938-1 |  |  |  |  |  |  |  |  |  |  |  |  |  |  |  |  |  |  |
|  | Pokkali | IRGC 8948-1 |  |  |  |  |  |  |  |  |  |  |  |  |  |  |  |  |  |  |
|  | Puttu nellu | IRGC 55346-1 |  |  |  |  |  |  |  |  |  |  |  |  |  |  |  |  |  |  |
|  | Rangoon Samba | IRGC 11940-1 |  |  |  |  |  |  |  |  |  |  |  |  |  |  |  |  |  |  |
|  | Race perumal | IRGC 55347-1 |  |  |  |  |  |  |  |  |  |  |  |  |  |  |  |  |  |  |
|  | Ranruwan | IRGC 36360-1 |  |  |  |  |  |  |  |  |  |  |  |  |  |  |  |  |  |  |
|  | Samba | IRGC 11993-1 |  |  |  |  |  |  |  |  |  |  |  |  |  |  |  |  |  |  |
|  | Sinna Sithira Kali | IRGC 51064-1 |  |  |  |  |  |  |  |  |  |  |  |  |  |  |  |  |  |  |
|  | Sigardis | IRGC 15555-1 |  |  |  |  |  |  |  |  |  |  |  |  |  |  |  |  |  |  |
|  | Sayam | IRGC 31538-1 |  |  |  |  |  |  |  |  |  |  |  |  |  |  |  |  |  |  |
|  | Sithaiyan Kottai Samba | IRGC 50155-1 |  |  |  |  |  |  |  |  |  |  |  |  |  |  |  |  |  |  |
|  | Sudu Karayal | IRGC 15348-1 |  |  |  |  |  |  |  |  |  |  |  |  |  |  |  |  |  |  |
|  | Vellai Kolomban | IRGC 15517-1 |  |  |  |  |  |  |  |  |  |  |  |  |  |  |  |  |  |  |
|  | WIR 1391 | IRGC 51605-1 |  |  |  |  |  |  |  |  |  |  |  |  |  |  |  |  |  |  |
|  | 105 | IRGC 40896-1 |  |  |  |  |  |  |  |  |  |  |  |  |  |  |  |  |  |  |
|  | 3210 | IRGC 116950-1 |  |  |  |  |  |  |  |  |  |  |  |  |  |  |  |  |  |  |
|  | A 69-1 | IRGC 55305-1 |  |  |  |  |  |  |  |  |  |  |  |  |  |  |  |  |  |  |
|  | BW 295-5 | IRGC 63098-1 |  |  |  |  |  |  |  |  |  |  |  |  |  |  |  |  |  |  |
|  | H6 | IRGC 157-1 |  |  |  |  |  |  |  |  |  |  |  |  |  |  |  |  |  |  |

Supplementary table 3: The presence of mutation in the exon 2 of Pi-ta gene

| **No** | **Name** | **Accession No** | 22 | 36 | 56 | 129 | 340 | 496 | 511 | 618 | 656 | 858 | 931 | 1045 | 1054 | 1077 | 1352 | 1359 | 1391 | 1579 | 1605 | 1609 | 1632 |
| --- | --- | --- | --- | --- | --- | --- | --- | --- | --- | --- | --- | --- | --- | --- | --- | --- | --- | --- | --- | --- | --- | --- | --- |
| 1. 1 | Alagusamba | IRGC 8944-2 |  |  |  |  |  |  |  |  |  |  |  |  |  |  |  |  |  |  |  |  |  |
| 1. 2 | Balasuriya | IRGC 66509-1 |  |  |  |  |  |  |  |  |  |  |  |  |  |  |  |  |  |  |  |  |  |
|  | Chandina | IRGC 36420-1 |  |  |  |  |  |  |  |  |  |  |  |  |  |  |  |  |  |  |  |  |  |
|  | Galawaka handeran | IRGC 31381-1 |  |  |  |  |  |  |  |  |  |  |  |  |  |  |  |  |  |  |  |  |  |
|  | Godawel | IRGC 15750-1 |  |  |  |  |  |  |  |  |  |  |  |  |  |  |  |  |  |  |  |  |  |
|  | Halsuduheenati | IRGC 15599-1 |  |  |  |  |  |  |  |  |  |  |  |  |  |  |  |  |  |  |  |  |  |
|  | Heendikwee | IRGC 15587-2 |  |  |  |  |  |  |  |  |  |  |  |  |  |  |  |  |  |  |  |  |  |
|  | Herath Banda | IRGC 67630-1 |  |  |  |  |  |  |  |  |  |  |  |  |  |  |  |  |  |  |  |  |  |
|  | Honderawala | IRGC 47372-1 |  |  |  |  |  |  |  |  |  |  |  |  |  |  |  |  |  |  |  |  |  |
|  | Hodarawala | IRGC 67631-1 |  |  |  |  |  |  |  |  |  |  |  |  |  |  |  |  |  |  |  |  |  |
|  | Kahatawee | IRGC 12004-1 |  |  |  |  |  |  |  |  |  |  |  |  |  |  |  |  |  |  |  |  |  |
|  | Kalu Ilankalayan | IRGC 36270-1 |  |  |  |  |  |  |  |  |  |  |  |  |  |  |  |  |  |  |  |  |  |
|  | Karutha Seenati | IRGC 15515-2 |  |  |  |  |  |  |  |  |  |  |  |  |  |  |  |  |  |  |  |  |  |
|  | Kotteyaran | IRGC 47383-1 |  |  |  |  |  |  |  |  |  |  |  |  |  |  |  |  |  |  |  |  |  |
|  | Kula karuppan | IRGC 55328-1 |  |  |  |  |  |  |  |  |  |  |  |  |  |  |  |  |  |  |  |  |  |
|  | Kurkaruppan | IRGC 15449-1 |  |  |  |  |  |  |  |  |  |  |  |  |  |  |  |  |  |  |  |  |  |
|  | Kurulu wee (white) | IRGC 66518-1 |  |  |  |  |  |  |  |  |  |  |  |  |  |  |  |  |  |  |  |  |  |
|  | Kurulutudu | IRGC 36304-1 |  |  |  |  |  |  |  |  |  |  |  |  |  |  |  |  |  |  |  |  |  |
|  | Matholuwa | IRGC 8901-1 |  |  |  |  |  |  |  |  |  |  |  |  |  |  |  |  |  |  |  |  |  |
|  | Moddai karuppan | IRGC 15465-1 |  |  |  |  |  |  |  |  |  |  |  |  |  |  |  |  |  |  |  |  |  |
|  | Murunga | IRGC15428-1 |  |  |  |  |  |  |  |  |  |  |  |  |  |  |  |  |  |  |  |  |  |
|  | Mudalige wee | IRGC 74706-1 |  |  |  |  |  |  |  |  |  |  |  |  |  |  |  |  |  |  |  |  |  |
|  | Muttu Samba | IRGC 36333-1 |  |  |  |  |  |  |  |  |  |  |  |  |  |  |  |  |  |  |  |  |  |
|  | Nalumoolai Karuppan | IRGC 8993-1 |  |  |  |  |  |  |  |  |  |  |  |  |  |  |  |  |  |  |  |  |  |
|  | Pachchaperumal | IRGC 3474-1 |  |  |  |  |  |  |  |  |  |  |  |  |  |  |  |  |  |  |  |  |  |
|  | Periya Vellai | IRGC 15475-1 |  |  |  |  |  |  |  |  |  |  |  |  |  |  |  |  |  |  |  |  |  |
|  | Podi heenati | IRGC 36345-1 |  |  |  |  |  |  |  |  |  |  |  |  |  |  |  |  |  |  |  |  |  |
|  | Pannithi | IRGC 51049-1 |  |  |  |  |  |  |  |  |  |  |  |  |  |  |  |  |  |  |  |  |  |
|  | Podiwee | IRGC 11938-1 |  |  |  |  |  |  |  |  |  |  |  |  |  |  |  |  |  |  |  |  |  |
|  | Pokkali | IRGC 8948-1 |  |  |  |  |  |  |  |  |  |  |  |  |  |  |  |  |  |  |  |  |  |
|  | Puttu nellu | IRGC 55346-1 |  |  |  |  |  |  |  |  |  |  |  |  |  |  |  |  |  |  |  |  |  |
|  | Rangoon Samba | IRGC 11940-1 |  |  |  |  |  |  |  |  |  |  |  |  |  |  |  |  |  |  |  |  |  |
|  | Race perumal | IRGC 55347-1 |  |  |  |  |  |  |  |  |  |  |  |  |  |  |  |  |  |  |  |  |  |
|  | Ranruwan | IRGC 36360-1 |  |  |  |  |  |  |  |  |  |  |  |  |  |  |  |  |  |  |  |  |  |
|  | Samba | IRGC 11993-1 |  |  |  |  |  |  |  |  |  |  |  |  |  |  |  |  |  |  |  |  |  |
|  | Sinna Sithira Kali | IRGC 51064-1 |  |  |  |  |  |  |  |  |  |  |  |  |  |  |  |  |  |  |  |  |  |
|  | Sigardis | IRGC 15555-1 |  |  |  |  |  |  |  |  |  |  |  |  |  |  |  |  |  |  |  |  |  |
|  | Sayam | IRGC 31538-1 |  |  |  |  |  |  |  |  |  |  |  |  |  |  |  |  |  |  |  |  |  |
|  | Sithaiyan Kottai Samba | IRGC 50155-1 |  |  |  |  |  |  |  |  |  |  |  |  |  |  |  |  |  |  |  |  |  |
|  | Sudu Karayal | IRGC 15348-1 |  |  |  |  |  |  |  |  |  |  |  |  |  |  |  |  |  |  |  |  |  |
|  | Vellai Kolomban | IRGC 15517-1 |  |  |  |  |  |  |  |  |  |  |  |  |  |  |  |  |  |  |  |  |  |
|  | WIR 1391 | IRGC 51605-1 |  |  |  |  |  |  |  |  |  |  |  |  |  |  |  |  |  |  |  |  |  |
|  | 105 | IRGC 40896-1 |  |  |  |  |  |  |  |  |  |  |  |  |  |  |  |  |  |  |  |  |  |
|  | 3210 | IRGC 116950-1 |  |  |  |  |  |  |  |  |  |  |  |  |  |  |  |  |  |  |  |  |  |
|  | A 69-1 | IRGC 55305-1 |  |  |  |  |  |  |  |  |  |  |  |  |  |  |  |  |  |  |  |  |  |
|  | BW 295-5 | IRGC 63098-1 |  |  |  |  |  |  |  |  |  |  |  |  |  |  |  |  |  |  |  |  |  |
|  | H6 | IRGC 157-1 |  |  |  |  |  |  |  |  |  |  |  |  |  |  |  |  |  |  |  |  |  |
