# Supplementary material for: In silico molecular and morphological analysis of rice blast resistant gene Pi-ta in Sri Lankan rice germplasm
Source: J Genet Eng Biotechnol. 2021 Oct 21;19:163. doi: 10.1186/s43141-021-00239-7 (PMC8531186; doi:10.1186/s43141-021-00239-7)
Supplement: Supplementary file 4 — Supplementary Table 3. Summary of mutation in the Pi-ta protein of 47 Sri Lankan rice accessions [file 43141_2021_239_MOESM4_ESM.docx]

| **Supplementary Table 3**: Summary of mutation in the Pi-ta protein of 47 Sri Lankan rice accessions | | | | | | | |
| --- | --- | --- | --- | --- | --- | --- | --- |
| No | Varieties | Accession No | Status of Mutation | Position | Original | Changed | Number of ORF |
| 1 | Alagusamba | IRGC 8944-2 | 0 |  |  |  | 22 |
| 2 | Balasuriya | IRGC 66509-1 | 3 | 6 | I | S | 22 |
|  |  |  |  | 118 | G | S |  |
|  |  |  |  | 918 | A | S |  |
| 3 | Chandina | IRGC 36420-1 | 2 | 6 | I | S | 22 |
|  |  |  |  | 918 | A | S |  |
| 4 | Galawaka handeran | IRGC 31381-1 | 2 | 6 | I | S | 22 |
|  |  |  |  | 918 | A | S |  |
| 5 | Godawel | IRGC 15750-1 | 5 | 6 | I | S | 22 |
|  |  |  |  | 148 | R | S |  |
|  |  |  |  | 158 | H | Q |  |
|  |  |  |  | 176 | D | V |  |
|  |  |  |  | 918 | A | S |  |
| 6 | Halsuduheenati | IRGC 15599-1 | 2 | 6 | I | S | 22 |
|  |  |  |  | 918 | A | S |  |
| 7 | Heendikwee | IRGC 15587-2 | 2 | 6 | I | S | 22 |
|  |  |  |  | 918 | A | S |  |
| 8 | Herath Banda | IRGC 67630-1 | 3 | 6 | I | S | 22 |
|  |  |  |  | 118 | G | S |  |
|  |  |  |  | 918 | A | S |  |
| 9 | Honderawala | IRGC 47372-1 | 0 |  |  |  | 22 |
| 10 | Hodarawala | IRGC 67631-1 | 19 | 6 | I | S | 20 |
|  |  |  |  | 79 | A | V |  |
|  |  |  |  | 162 | H | D |  |
|  |  |  |  | 230 | K | R |  |
|  |  |  |  | 234 | I | V |  |
|  |  |  |  | 315 | R | K |  |
|  |  |  |  | 386 | V | F |  |
|  |  |  |  | 395 | H | Y |  |
|  |  |  |  | 403 | M | I |  |
|  |  |  |  | 466 | H | R |  |
|  |  |  |  | 479 | K | R |  |
|  |  |  |  | 571 | A | S |  |
|  |  |  |  | 644 | L | I |  |
|  |  |  |  | 711 | T | N |  |
|  |  |  |  | 724 | R | C |  |
|  |  |  |  | 816 | L | F |  |
|  |  |  |  | 887 | H | N |  |
|  |  |  |  | 911 | P | L |  |
|  |  |  |  | 918 | A | S |  |
| 11 | Kahatawee | IRGC 12004-1 | 2 | 6 | I | S | 22 |
|  |  |  |  | 918 | A | S |  |
| 12 | Kalu Ilankalayan | IRGC 36270-1 | 2 | 6 | I | S | 22 |
|  |  |  |  | 918 | A | S |  |
| 13 | Karutha Seenati | IRGC 15515-2 | 19 | 6 | I | S | 20 |
|  |  |  |  | 79 | A | V |  |
|  |  |  |  | 162 | H | D |  |
|  |  |  |  | 230 | K | R |  |
|  |  |  |  | 234 | I | V |  |
|  |  |  |  | 315 | R | K |  |
|  |  |  |  | 386 | V | F |  |
|  |  |  |  | 395 | H | Y |  |
|  |  |  |  | 403 | M | I |  |
|  |  |  |  | 466 | H | R |  |
|  |  |  |  | 479 | K | R |  |
|  |  |  |  | 571 | A | S |  |
|  |  |  |  | 644 | L | I |  |
|  |  |  |  | 711 | T | N |  |
|  |  |  |  | 724 | R | C |  |
|  |  |  |  | 816 | L | F |  |
|  |  |  |  | 887 | H | N |  |
|  |  |  |  | 911 | P | L |  |
|  |  |  |  | 918 | A | S |  |
| 14 | Kotteyaran | IRGC 47383-1 | 2 | 6 | I | S | 22 |
|  |  |  |  | 918 | A | S |  |
| 15 | Kula karuppan | IRGC 55328-1 | 2 | 6 | I | S | 22 |
|  |  |  |  | 918 | A | S |  |
| 16 | Kurkaruppan | IRGC 15449-1 | 2 | 6 | I | S | 22 |
|  |  |  |  | 918 | A | S |  |
| 17 | Kurulu wee (white) | IRGC 66518-1 | 1 | 918 | A | S | 22 |
| 18 | Kurulutudu | IRGC 36304-1 | 2 | 6 | I | S | 22 |
|  |  |  |  | 918 | A | S |  |
| 19 | Matholuwa | IRGC 8901-1 | 19 | 6 | I | S | 20 |
|  |  |  |  | 79 | A | V |  |
|  |  |  |  | 162 | H | D |  |
|  |  |  |  | 230 | K | R |  |
|  |  |  |  | 234 | I | V |  |
|  |  |  |  | 315 | R | K |  |
|  |  |  |  | 386 | V | F |  |
|  |  |  |  | 395 | H | Y |  |
|  |  |  |  | 403 | M | I |  |
|  |  |  |  | 466 | H | R |  |
|  |  |  |  | 479 | K | R |  |
|  |  |  |  | 571 | A | S |  |
|  |  |  |  | 644 | L | I |  |
|  |  |  |  | 711 | T | N |  |
|  |  |  |  | 724 | R | C |  |
|  |  |  |  | 816 | L | F |  |
|  |  |  |  | 887 | H | N |  |
|  |  |  |  | 911 | P | L |  |
|  |  |  |  | 918 | A | S |  |
| 20 | Moddai karuppan | IRGC 15465-1 | 2 | 6 | I | S | 22 |
|  |  |  |  | 918 | A | S |  |
| 21 | Murunga | IRGC15428-1 | 2 | 6 | I | S | 22 |
|  |  |  |  | 918 | A | S |  |
| 22 | Mudalige wee | IRGC 74706-1 | 18 | 6 | I | S | 20 |
|  |  |  |  | 79 | A | V |  |
|  |  |  |  | 162 | H | D |  |
|  |  |  |  | 230 | K | R |  |
|  |  |  |  | 234 | I | V |  |
|  |  |  |  | 315 | R | K |  |
|  |  |  |  | 386 | V | F |  |
|  |  |  |  | 395 | H | Y |  |
|  |  |  |  | 403 | M | I |  |
|  |  |  |  | 479 | K | R |  |
|  |  |  |  | 571 | A | S |  |
|  |  |  |  | 644 | L | I |  |
|  |  |  |  | 711 | T | N |  |
|  |  |  |  | 725 | R | C |  |
|  |  |  |  | 816 | L | F |  |
|  |  |  |  | 887 | H | N |  |
|  |  |  |  | 911 | P | L |  |
|  |  |  |  | 918 | A | S |  |
| 23 | Muttu Samba | IRGC 36333-1 | 2 | 6 | I | S | 22 |
|  |  |  |  | 918 | A | S |  |
| 24 | Nalumoolai Karuppan | IRGC 8993-1 | 2 | 6 | I | S | 22 |
|  |  |  |  | 918 | A | S |  |
| 25 | Pachchaperumal | IRGC 3474-1 | 0 |  |  |  | 22 |
| 26 | Periya Vellai | IRGC 15475-1 | 2 | 6 | I | S | 22 |
|  |  |  |  | 918 | A | S |  |
| 27 | Podi heenati | IRGC 36345-1 | 19 | 6 | I | S | 20 |
|  |  |  |  | 79 | A | V |  |
|  |  |  |  | 162 | H | D |  |
|  |  |  |  | 230 | K | R |  |
|  |  |  |  | 234 | I | V |  |
|  |  |  |  | 315 | R | K |  |
|  |  |  |  | 386 | V | F |  |
|  |  |  |  | 395 | H | Y |  |
|  |  |  |  | 403 | M | I |  |
|  |  |  |  | 466 | H | R |  |
|  |  |  |  | 479 | K | R |  |
|  |  |  |  | 571 | A | S |  |
|  |  |  |  | 644 | L | I |  |
|  |  |  |  | 711 | T | N |  |
|  |  |  |  | 724 | R | C |  |
|  |  |  |  | 816 | L | F |  |
|  |  |  |  | 887 | H | N |  |
|  |  |  |  | 911 | P | L |  |
|  |  |  |  | 918 | A | S |  |
| 28 | Pannithi | IRGC 51049-1 | 3 | 6 | I | S | 22 |
|  |  |  |  | 118 | G | S |  |
|  |  |  |  | 918 | A | S |  |
| 29 | Podiwee | IRGC 11938-1 | 0 |  |  |  | 22 |
| 30 | Pokkali | IRGC 8948-1 | 0 |  |  |  | 22 |
| 31 | Puttu nellu | IRGC 55346-1 | 3 | 6 | I | S | 22 |
|  |  |  |  | 118 | G | S |  |
|  |  |  |  | 918 | A | S |  |
| 32 | Rangoon Samba | IRGC 11940-1 | 3 | 6 | I | S | 22 |
|  |  |  |  | 118 | G | S |  |
|  |  |  |  | 918 | A | S |  |
| 33 | Race perumal | IRGC 55347-1 | 0 |  |  |  | 22 |
| 34 | Ranruwan | IRGC 36360-1 | 19 | 6 | I | S | 20 |
|  |  |  |  | 79 | A | V |  |
|  |  |  |  | 162 | H | D |  |
|  |  |  |  | 230 | K | R |  |
|  |  |  |  | 234 | I | V |  |
|  |  |  |  | 315 | R | K |  |
|  |  |  |  | 386 | V | F |  |
|  |  |  |  | 395 | H | Y |  |
|  |  |  |  | 403 | M | I |  |
|  |  |  |  | 466 | H | R |  |
|  |  |  |  | 479 | K | R |  |
|  |  |  |  | 571 | A | S |  |
|  |  |  |  | 644 | L | I |  |
|  |  |  |  | 711 | T | N |  |
|  |  |  |  | 724 | R | C |  |
|  |  |  |  | 816 | L | F |  |
|  |  |  |  | 887 | H | N |  |
|  |  |  |  | 911 | P | L |  |
|  |  |  |  | 918 | A | S |  |
| 35 | Samba | IRGC 11993-1 | 0 |  |  |  | 22 |
| 36 | Sinna Sithira Kali | IRGC 51064-1 | 3 | 6 | I | S | 22 |
|  |  |  |  | 118 | G | S |  |
|  |  |  |  | 918 | A | S |  |
| 37 | Sigardis | IRGC 15555-1 | 2 | 6 | I | S | 22 |
|  |  |  |  | 918 | A | S |  |
| 38 | Sayam | IRGC 31538-1 | 17 | 6 | I | S | 20 |
|  |  |  |  | 79 | A | V |  |
|  |  |  |  | 162 | H | D |  |
|  |  |  |  | 230 | K | R |  |
|  |  |  |  | 234 | I | V |  |
|  |  |  |  | 315 | R | K |  |
|  |  |  |  | 386 | V | F |  |
|  |  |  |  | 395 | H | Y |  |
|  |  |  |  | 403 | M | I |  |
|  |  |  |  | 466 | H | R |  |
|  |  |  |  | 571 | A | S |  |
|  |  |  |  | 644 | L | I |  |
|  |  |  |  | 711 | T | N |  |
|  |  |  |  | 816 | L | F |  |
|  |  |  |  | 887 | H | N |  |
|  |  |  |  | 911 | P | L |  |
|  |  |  |  | 918 | A | S |  |
| 39 | Sithaiyan Kottai Samba | IRGC 50155-1 | 0 |  |  |  | 22 |
| 40 | Sudu Karayal | IRGC 15348-1 | 2 | 6 | I | S | 22 |
|  |  |  |  | 918 | A | S |  |
| 41 | Vellai Kolomban | IRGC 15517-1 | 2 | 6 | I | S | 22 |
|  |  |  |  | 918 | A | S |  |
| 42 | WIR 1391 | IRGC 51605-1 | 1 | 918 | A | S | 22 |
| 43 | 105 | IRGC 40896-1 | 3 | 6 | I | S | 22 |
|  |  |  |  | 118 | G | S |  |
|  |  |  |  | 918 | A | S |  |
| 44 | 3210 | IRGC 116950-1 | 0 |  |  |  | 22 |
| 45 | A 69-1 | IRGC 55305-1 | 19 | 6 | I | S | 20 |
|  |  |  |  | 18 | S | T |  |
|  |  |  |  | 66 | E | Q |  |
|  |  |  |  | 79 | A | V |  |
|  |  |  |  | 162 | H | D |  |
|  |  |  |  | 230 | K | R |  |
|  |  |  |  | 234 | I | V |  |
|  |  |  |  | 315 | R | K |  |
|  |  |  |  | 395 | H | Y |  |
|  |  |  |  | 403 | M | I |  |
|  |  |  |  | 466 | H | R |  |
|  |  |  |  | 571 | A | S |  |
|  |  |  |  | 644 | L | I |  |
|  |  |  |  | 711 | T | N |  |
|  |  |  |  | 724 | R | C |  |
|  |  |  |  | 816 | L | F |  |
|  |  |  |  | 887 | H | N |  |
|  |  |  |  | 911 | P | L |  |
|  |  |  |  | 918 | A | S |  |
| 46 | BW 295-5 | IRGC 63098-1 | 0 |  |  |  | 22 |
| 47 | H6 | IRGC 157-1 | 2 | 6 | I | S | 22 |
|  |  |  |  | 918 | A | S |  |
