# Supplementary material for: In silico molecular and morphological analysis of rice blast resistant gene Pi-ta in Sri Lankan rice germplasm
Source: J Genet Eng Biotechnol. 2021 Oct 21;19:163. doi: 10.1186/s43141-021-00239-7 (PMC8531186; doi:10.1186/s43141-021-00239-7)
Supplement: Supplementary file 5 — Supplementary table 4. List of 47 Sri Lankan rice accessions deposited in Rice SNP-Seek Database of the International Rice Research Institute [file 43141_2021_239_MOESM5_ESM.docx]

Supplementary table 4: List of 47 Sri Lankan rice accessions deposited in Rice SNP-Seek Database of the International Rice Research Institute

| **No.** | **Accession name** | **IRGC Number** |
| --- | --- | --- |
| 1 | Alagusamba | IRGC 8944-2 |
| 2 | Balasuriya | IRGC 66509-1 |
| 3 | Chandina | IRGC 36420-1 |
| 4 | Galawaka handeran | IRGC 31381-1 |
| 5 | Godawel | IRGC 15750 |
| 6 | Halsuduheenati | IRGC 15599-1 |
| 7 | Heendikwee | IRGC 15587-2 |
| 8 | Herath Banda | IRGC 67630-1 |
| 9 | Honderawala | IRGC 47372-1 |
| 10 | Hodarawala | IRGC 67631-1 |
| 11 | Kahatawee | IRGC 12004-1 |
| 12 | Kalu Ilankayan | IRGC 36270-1 |
| 13 | Karutha seenati | IRGC 15515-2 |
| 14 | Kotteyaran | IRGC 47383-1 |
| 15 | Kula karupan | IRGC 55328-1 |
| 16 | Kurkaruppan | IRGC 15449-1 |
| 17 | Kurulu wee (White) | IRGC 66518-1 |
| 18 | Kurulutudu | IRGC 36304-1 |
| 19 | Matholuwa | IRGC 8901-1 |
| 20 | Moddai karupan | IRGC 15465-1 |
| 21 | Murunga | IRGC 15428-1 |
| 22 | Mudalige wee | IRGC 74706-1 |
| 23 | Muttu Samba | IRGC 36333-1 |
| 24 | Nalumoolaikarupan | IRGC 8993-1 |
| 25 | Pachchaperumal | IRGC 3474-1 |
| 26 | Periya vellai | IRGC 15475-1 |
| 27 | Podi heenati | IRGC 36345-1 |
| 28 | Pannithi | IRGC 51049-1 |
| 29 | Podiwee | IRGC 11938-1 |
| 30 | Pokkali | IRGC 8948-1 |
| 31 | Puttu nellu | IRGC 55346-1 |
| 32 | Rangoon samba | IRGC 11940-1 |
| 33 | Race perumal | IRGC 55347-1 |
| 34 | Ranruwan | IRGC 36360-1 |
| 35 | Samba | IRGC 11993-1 |
| 36 | Sinna sithira kalli | IRGC 51064-1 |
| 37 | Sigardis | IRGC 15555-1 |
| 38 | Sayam | IRGC 31538-1 |
| 39 | Sithaiyankottai samba | IRGC 50155-1 |
| 40 | Sudu karayal | IRGC 15348-1 |
| 41 | Vellai kollumban | IRGC 15517-1 |
| 42 | WIR 1391 | IRGC 51605-1 |
| 43 | 105 | IRGC 40896-1 |
| 44 | 3210 | IRGC 1116950-1 |
| 45 | A69-1 | IRGC 55305-1 |
| 46 | Bw295-5 | IRGC 63098-1 |
| 47 | H6 | IRGC 157-1 |

IRGC, International Rice Genebank Collection
